# Biomimetic Growth of Hydroxyapatite on SiO_2_ Microspheres to Improve Its Biocompatibility and Gentamicin Loading Capacity

**DOI:** 10.3390/ma14226941

**Published:** 2021-11-17

**Authors:** Alejandra E. Herrera-Alonso, María C. Ibarra-Alonso, Sandra C. Esparza-González, Sofía Estrada-Flores, Luis A. García-Cerda, Antonia Martínez-Luévanos

**Affiliations:** 1 Department of Advanced Ceramic Materials and Energy, Facultad de Ciencias Químicas, Universidad Autónoma de Coahuila, Blvd. V. Carranza s/n, Saltillo 25280, Coahuila, Mexico; alejandra_herrera_alonso@uadec.edu.mx (A.E.H.-A.); sofiaestrada@uadec.edu.mx (S.E.-F.); 2 School of Chemical Sciences, CONACYT-Universidad Autónoma de Coahuila, Blvd. V. Carranza s/n, Saltillo 25280, Coahuila, Mexico; 3 Facultad de Odontología, Universidad Autónoma de Coahuila, Saltillo 25280, Coahuila, Mexico; sandraesparzagonzal@uadec.edu.mx; 4 Centro de Investigación en Química Aplicada, Blvd. Enrique Reyna, Hermosillo 140, San José de los Cerritos, Saltillo 25294, Coahuila, Mexico; luis.garcia@ciqa.edu.mx

**Keywords:** biocompatibility, biomimetic growth, gentamicin, drug load, hydroxyapatite, silica

## Abstract

The interest in multifunctional biomaterials to be implanted are also able to release drugs that reduce pain and inflammation or prevent a possible infection has increased. Bioactive materials such as silica (SiO_2_) containing surface silanol groups contribute to the nucleation and growth of hydroxyapatite (HAp) in a physiological environment. Regarding biocompatibility, the spherical shape of particles is the desirable one, since it does not cause mechanical damage to the cell membrane. In this work, the synthesis of SiO_2_ microspheres was performed by the modified Stöber method and they were used for the biomimetic growth of HAp on their surface. The effect of the type of surfactant (sodium dodecyl sulphate (SDS), cetyltrimethylammonium bromide (CTAB), and polyethylene glycol (PEG)), and heat treatment on the morphology and size of SiO_2_ particles was investigated. Monodisperse, spherical-shaped SiO_2_ microparticles with an average particle size of 179 nm, were obtained when using PEG (SiO_2_-PEG). The biomimetic growth of HAp was performed on this sample to improve its biocompatibility and drug-loading capacity using gentamicin as a model drug. Biomimetic growth of HAp was confirmed by FTIR-ATR, SEM-EDX and TEM techniques. SiO_2_-PEG/HAp sample had a better biocompatibility in vitro and gentamicin loading capacity than SiO_2_-PEG sample.

## 1. Introduction

Bone is the only part of the body with the capacity to regenerate, this bone regeneration generally requires three processes: osteoconduction (in which the graft material provides an appropriate physical environment for new bone creation), osteoinduction (encourages active osteoblasts to stimulate osteogenesis), and osteogenesis (new bone formation from cells derived from the graft or the host) [1,2]. When there are bone problems, bone is naturally removed and new bone tissue is synthesized based on phosphates, calcium carbonates, collagen, and proteins [2,3], however, in advanced age, this process is negatively affected [4,5]. Hydroxyapatite made up of crystalline calcium phosphates facilitates bone regeneration due to its similarity in composition to human bone and it has an excellent biocompatibility [3,6]. In addition to hydroxyapatite for its use in bone tissue engineering, extensive research has also been carried out on bioglass and SiO_2_. On the other hand, for a decade interest has been increasing in multifunctional biomaterials that besides being implanted are also able to release drugs that reduce pain and inflammation or prevent a possible infection.

Our interest in SiO_2_ microspheres focuses on their ability to induce HAp biomimetic growth [7,8,9]. Silanol groups (Si–OH) on the surface of SiO_2_ provide effective sites for nucleation and biomimetic growth of HAp, which has been widely reported to be biocompatible [10,11,12,13]. In a simulated body fluid (SBF), with plenty of Ca^2+^ and PO_4_^3−^ ions, HAp formation can be explained by the presence of Si–OH groups. Silanol (Si–OH) can bind to the Ca^2+^ ions present in the SBF solution, forming a calcium silicate and also providing a positive charge on the silica surface, which will attract PO_4_^3−^ groups giving rise to calcium phosphate. HAp nuclei formation is facilitated with this process, which occurs spontaneously, consuming Ca^2+^ and PO_4_^3−^ ions present in SBF solution [7,12,13].

The synthesis of SiO_2_ microspheres by the Stöber method and using various surfactants as templates to employ them in various applications have been previously reported [14,15,16]. However, the use of surfactants during SiO_2_ synthesis to control shape and size may affect biocompatibility, even in trace amounts, since most surfactants are cytotoxic [17,18,19]. On the other hand, the use of SiO_2_ and HAp in tissue engineering has been widely reported. For a decade, interest in multifunctional biomaterials has been increasing. It is desirable that this type of biomaterial, to be implanted, have the capacity to release drugs that help to reduce pain and inflammation, or to prevent a possible infection.

The aim of this work is to obtain spherical SiO_2_ microparticles by the modified Stöber method, using SDS, CTAB or PEG surfactants as templates, with the presence of the Si–OH group on its surface to facilitate the biomimetic growth of hydroxyapatite (HAp), with the purpose of improving its biocompatibility and gentamicin loading capacity.

## 2. Materials and Methods

### 2.1. Materials

The reagents used for the synthesis of spherical silica microparticles were purchased from Aldrich and used without further treatment; tetraethylorthosilicate (SiC_8_H_20_O_4_) (TEOS, 98%, Aldrich, Mexico, Mexico), cetyltrimethylammonium bromide (C_19_H_42_BrN, CTAB, 99%, Aldrich), sodium dodecyl sulfate (NaC_12_H_25_SO_4_, SDS, 99%, Aldrich) and ammonium hydroxide (NH_4_OH, 98%, Aldrich). Polyethylene glycol was also purchased (PEG, M.W. 1000, Aldrich) and anhydrous ethanol with 99% purity (Jalmek, Nuevo Leon, Mexico). The reagent (3-(4,5-dimethylthiazol-2-yl)-2,5-diphenyltetrazolium bromide ((MTT), Aldrich) was used for the cytotoxicity tests.

### 2.2. Methods

#### 2.2.1. Synthesis of SiO_2_ Microspheres: Effect of Surfactant and Heat Treatment

The SiO_2_ synthesis was performed using the modified Stöber method, which consists of hydrolysis and condensation of TEOS, a mixture of EtOH: H_2_O with a molar ratio of 5:1 was used. First, 0.0469 moles of NH_4_OH were added, which acts as a catalyst, then 0.005 moles of TEOS are added. Finally, the surfactant is added at a concentration 10 times the critical micellar concentration (CMC). SiO_2_ samples were synthesized using SDS, CTAB, and PEG surfactants as templates, to investigate their effect on morphology and to obtain spherical particles with sizes in the micrometric scale. SiO_2_ obtained samples were named SiO_2_-SDS, SiO_2_-CTAB y SiO_2_-PEG, respectively. The effect of the heat treatment at 600 °C for 6 h, with a heating rate of 1 °C/min, was also determined, in the morphology of the three SiO_2_ samples obtained. Subsequently, the best sample was selected to induce the biomimetic growth of HAp, as well as to carry out biocompatibility tests of hemolysis and cell viability.

#### 2.2.2. Biomimetic Growth of Hydroxyapatite on SiO_2_

SBF (simulated body fluid) solution was prepared as reported by Kokubo et al. [20]. Each SiO_2_ sample was soaked in 50 mL of SBF solutions at 37 °C and at pH of 7.4. Hemolysis tests were made at 3, 5 and 7 ppm of each silica sample. The propylene tubes containing the different dispersions were placed in a VWR Incubating Mini Shaker for 21 days. After the induction of biomimetic growth of HAp, the morphological characterization of the SiO_2_ samples was carried out using a JEOL JSM-7800F microscope operated at 10 kV.

#### 2.2.3. In Vitro Biocompatibility

##### Hemolysis Test

For the hemolysis test, a procedure established by ASTM F756-13 was followed [21]. Blood samples were briefly obtained in Vacutainer tubes with EDTA as an anticoagulant, they were subsequently centrifuged at 3000 rpm for 5 min at 5 °C, the plasma was discarded (supernatant) and the cell precipitate was washed three times with a saline solution called Alsever solution, to prevent blood clotting. We placed 3, 5, and 7 ppm of the samples with 150 µL of the erythrocyte solution diluted with 1850 µL of Alsever solution. The diluted erythrocyte solution and the deionized water were used as negative and positive control, respectively. The suspension of erythrocytes in the presence of the silica samples was kept at 37 °C for 24 h. At the end of this time, the suspension was centrifuged at 3000 rpm for 5 min at 5 °C. 1 mL of supernatant was taken to measure the absorbance at 415 nm in a UV-Vis-NIR spectrophotometer (Jenway, model 7305, Cole-Parmer, Staffordshire, UK). The hemolysis ratio (% H) was determined using the following equation:

(1) $$\%H=\frac{Abs\;\left(M\right)-Abs\;\left(CN\right)}{Abs\;\left(CP\right)-Abs\;\left(CN\right)}\times 100\%$$

where: *Abs* (*M*) = sample absorbance, *Abs* (*CN*) = negative control absorbance, *Abs* (*CP*) = positive control absorbance.

##### Cytotoxicity Using the Cell Line 3T3: MTT Test

The cytotoxicity test was based on the ISO 10993-5 standard procedure [22]. Mice fibroblast cell lines 3T3 was used. These cells were incubated with 3 mL of culture medium. Dulbecco’s modified Eagle medium (DMEM) supplemented with 10% of phosphate buffer solution (PBS), 1% HEPPES buffer ((4-(2-hydroxyethyl)-1-piperazineethanesulfonic acid)), 1% non-essential amino acids, 1% penicillin/streptomycin and 1% pyruvate at 37 °C and 5% of CO_2_ atmosphere for 5 days. Subsequently, the cells were seeded in a 96-well plate with 200 µL supplemented DMEM and a density of 7500 cells per well, allowed to stand for 24 h. The silica samples to be evaluated were prepared at a concentration of 3 and 5 ppm, using the same cell culture medium as a solvent. The culture medium was removed, and the samples were placed at different concentrations with at least 3 replicates per concentration. Samples were incubated again at 37 °C, with a 5% CO_2_ atmosphere for 24 h. We used 200 µL of medium as negative control and another 200 µL of dimethylsulfoxide (DMSO) as positive death control. After removing the culture medium, 20 μL of (3-(4,5-dimethylthiazol-2-yl)-2,5-diphenyltetrazolium bromide (MTT) was added to each culture well at the concentration of 5 mg/mL. The plates with cell culture and samples were incubated for 4 h at 37 °C and 5% CO_2_ atmosphere, then the medium was carefully removed and 100 μL of DMSO was added to dissolve formazan crystals and quantify the color. Absorbance values were read on a UV-Vis Thermoscientific Multiscan spectrophotometer at a wavelength of 575 nm. The percentage of cell viability of the samples was calculated using the following equation:

(2) $$\%\;Cell\;Viability=\frac{Abs_{\left(M\right)}}{Abs_{\left(CN\right)}{}_{\;}}\times 100\;\%$$

where: *Abs* (*M*) = simple absorbance, *Abs* (*CN*) = negative control absorbance.

A statistical analysis was performed using the Prism Graph 7.0 software, from the data obtained a one-way analysis of variance (ANOVA) was performed, and the importance of the experimental results with the controls was expressed (*p* ≤ 0.05).

#### 2.2.4. Gentamicin Loading Capacity

The gentamicin loading was carried out by the impregnation method, it consists of immersing 0.2 g of SiO_2_ and SiO_2_/HAp samples in 10 mL of a gentamicin solution of 20 mg/mL (pharmaceutical solution). These dispersions were performed in triplicate and were kept in an incubator at 37 °C for 24 h. After this time, dispersions were centrifuged to separate the liquid phase from the solid one, and the clear solutions were used to read the absorbance with a UV-Vis-NIR spectrophotometer (Jenway, model 7305) at a wavelength of 254 nm. Gentamicin concentration was calculated using a lineal equation that was previously obtained from a calibration curve of absorbance vs. gentamicin concentration. The following equation was used to determine the percentage of loading capacity (%):

(3) $$Loading\;Capacity\;\left(\%\right)=\frac{Gentamicin\;in\;the\;sample\;\left(mg\right)}{Sample\;\left(mg\right)}\times 100\%\;$$

#### 2.2.5. Characterization

SiO_2_ and SiO_2_/HAp samples were characterized by infrared spectroscopy (FTIR) with ATR accessory; Infrared spectra were recorded over a range of 400 to 4000 cm^−1^, with 100 scans and a resolution of 0.4 cm^−1^ using a spectrophotometer (Thermo Scientific-Nicolet, model iS10, Thermo Fisher Scientific, Waltham, Massachusetts, US). Morphologies of the samples were investigated with two field emission scanning electron microscopes, with energy-dispersive X-ray spectroscopy, EDX (Hitachi SU8010 and Jeol JSM-7800F), operated at a voltage of 1 to 14 kV. Before this analysis, SiO_2_ samples were coated with gold nanoparticles to facilitate electrical conductivity. The measurement of the particle diameter size was made with Image software, counting approximately 100 particles. SiO2/HAp sample was also characterized by a transmission electron microscopy technique (Jeol, JEM-2100). X-ray diffraction (XRD) patterns were obtained using a Rigaku Ultima IV with D-Tex detector, with Cu tube and Kα radiation at 1.5405 Å, scanning in the 5–80° (2 theta symbol range) with increments of 0.02° and a sweep time of 0.2 s, operated at 40 kV and 44 mA. The specific surface area of the samples was estimated according to the Brunauer–Emmet–Teller (BET) method using a physisorption equipment (Beckman Coulter SA 3100). Pore size distribution was calculated according to the model Barret–Joyner–Halenda (BJH). Size distribution and zeta potential were measured with Litesizer (Anton Paar, Graz, Austria) equipment, using laser dynamic scattering and microelectrophoresis techniques, respectively. Suspensions of the SiO_2_-PEG and SiO_2_-PEG/HAp samples were prepared at a concentration of 1 mg / ml. The ionic strength of the solution was adjusted to 1 mM with KCl.

## 3. Results and Discussion

### 3.1. Synthesis of SiO_2_ Microspheres: Effect of Surfactant and Heat Treatment

The results of the FTIR-ATR, SEM, and BET characterization of the silica samples are presented. The effect of the type of surfactant (SDS, CTAB, and PEG) and heat treatment on the morphology and size of SiO_2_ microspheres was investigated. Subsequently, the best sample was selected to induce the biomimetic growth of HAp and carry out the biocompatibility analysis, as well as the gentamicin load.

**Figure 1:**
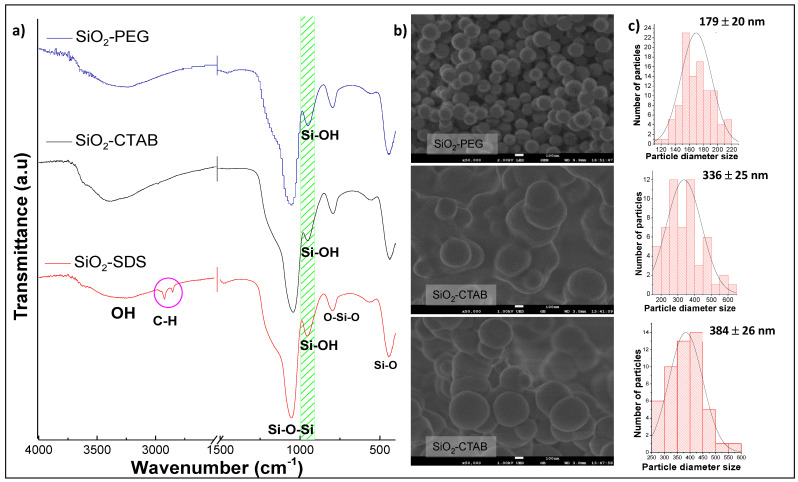
(**a**) Fourier transform infrared spectroscopy with attenuated total reflectance (FTIR-ATR) spectra and (**b**) micrographs at 50,000 X, and (**c**) particle size distribution of SiO_2_-PEG, SiO_2_-CTAB, andSiO_2_-SDS samples.

The characteristic absorption bands of silica are indicated in Table 1 [17]. The results of the infrared spectroscopy of the different samples analyzed are shown in Figure 1a, which presents the infrared spectra of the three silica samples obtained, varying the type of surfactant (PEG, CTAB, and SDS). The characteristic absorption bands of SiO_2_ can be observed, mainly the characteristic band of the silanol group (Si–OH) at 944 cm^−1^. FTIR spectrum of the SiO2-SDS sample show a small absorption band at 2926 cm^−1^, corresponding to stretching of the C-H bond, of the SDS hydrocarbon chain, indicating that this surfactant is still in the sample. However, the heat treatment is expected to remove traces of surfactant present in the SiO2-SDS sample.

Figure 1b shows the micrographs of SiO_2_-SDS, SiO_2_-CTAB, and SiO_2_-PEG samples; also, particle size distribution is shown. SiO_2_ samples are conformed by microparticles with spherical morphology. The concentration used for each surfactant was 10 times higher than the critical micellar concentration (CMC). Surfactant micelles acted as a template, surrounding the SiO_2_, providing the spherical morphology [23,24]. For SiO_2_-CTAB y SiO_2_-SDS samples, a spherical morphology was also shown; however, agglomeration of these spheres was obtained, with a larger average particle diameter size of 336 and 384 nm, respectively. The effect of agglomeration of SiO_2_ particles is due to the aggregation, according to the DLVO theory (Deryaguin–Landau–Verwey–Oberbeek) [25,26,27] collision between colloidal particles is caused by Brownian motion and the existence of attractive forces of the Van der Waals type. This effect was evident for the SiO_2_-CTAB and SiO_2_-SDS samples.

Liu et al. in 2013, propose that cationic surfactant CTAB cannot effectively coat the SiO_2_ spheres, due to the neutralization of the surface charge, between the positively charged surfactant groups (CTAB^+^) and siloxane groups (Si-O-), therefore, aggregation of nanoparticles was observed [28]. In using anionic surfactant SDS (SiO_2_-SDS), a lower degree of sphere aggregation is observed, compared to SiO_2_-CTAB. Guo-Yong et al. (2014) mention that the SDS surfactant is also a foaming agent, producing a large number of bubbles in the reaction solution, which make it difficult to control the size of the sphere [29].

For SiO_2_-PEG sample, fine spheres around 179 nm of average diameter size were observed. In general, the SiO_2_-PEG sample presented a homogeneous size and no aggregation of microparticles, attributed to better stability due to the use of non-ionic surfactant, resulting in better interaction with the particles avoiding aggregation and favoring smaller sizes compared to SiO_2_ obtained by SDS and CTAB surfactants (Figure 1c) [28,29,30,31].

**Figure 2:**
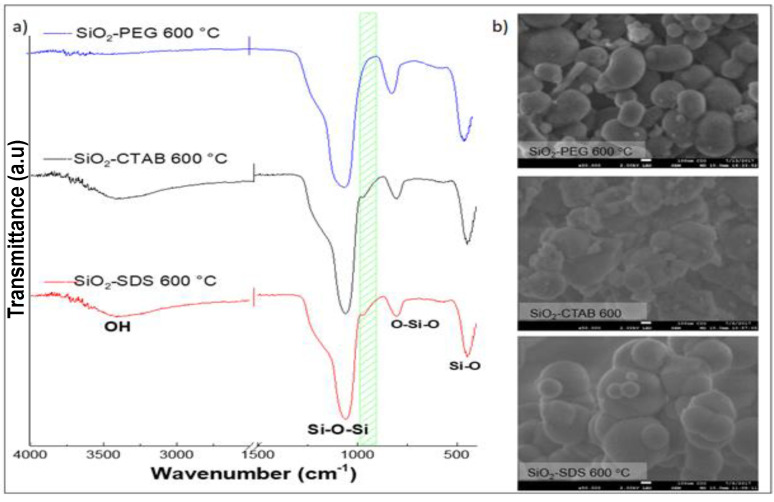
(**a**) FTIR-ATR spectra and (**b**) micrographs at 50,000 X of SiO_2_-PEG, SiO_2_-CTAB, and SiO_2_-SDS samples, heat-treated at 600 °C with a heating rate of 1 °C/min.

To eliminate the traces of surfactant that were used as templates and to generate more pores in the SiO_2_ samples, they were subjected to a heat treatment at 600 °C for 6 h. Figure 2a shows FTIR-ATR spectra of SiO_2_ samples that were synthesized with the three different surfactants (PEG, CTAB, and SDS), after heat treatment. The results showed the absence of surfactant traces, however, the characteristic bands of the silanol group (Si–OH), OH at 944 cm^−1^, and 3400 cm^−1^, respectively, also disappeared. Xianfeng Zhou et al. mention that the silanol group (Si–OH) acts as a nucleating agent for hydroxyapatite formation (HAp) [13]. Therefore, being a silanol group (Si–OH) decisive in bioactivity [7,10,13], we can predict that the samples after heat treatment will not be promising in the biomimetic growth of HAp. Micrographs of SiO_2_-SDS-600, SiO_2_-CTAB-600, and SiO_2_-PEG-600 samples are shown in Figure 2b. In all the samples an aggregation of the spheres was observed after carrying out heat treatment [30].

According to the results obtained, the heat treatment is ruled out, in the three samples of silica obtained, it is concluded that after the heat treatment, the spherical morphology collapses, besides, the silanol group (Si–OH) is eliminated in SiO_2_ samples (Figure 1b). Since silanol group is decisive for the nucleation and growth of hydroxyapatite (HAp), it is recommended not to treat SiO_2_ samples at 600 °C.

**Figure 3:**
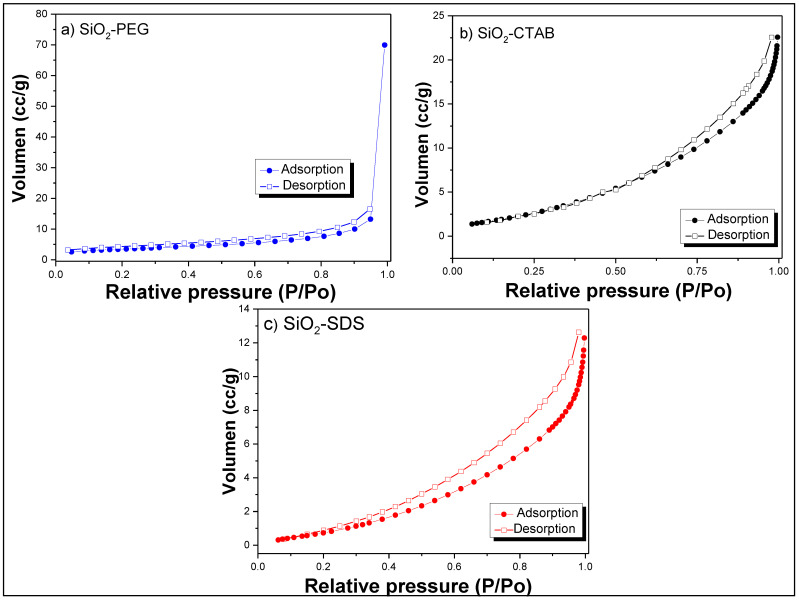
Adsorption-desorption isotherms of (**a**) SiO_2_-SDS, (**b**) SiO_2_-CTAB and (**c**) SiO_2_-PEG.

Figure 3 shows the adsorption-desorption isotherms of SiO_2_ samples that were obtained without heat treatment. The adsorption isotherm of the SiO_2_-PEG sample is type III (Figure 3a) with a H_4_ type hysteresis loop, whereas SiO_2_-CTAB and SiO_2_-SDS samples presented a mixture between type III and IV (Figure 3b,c, respectively) with a H_3_ type hysteresis loop, indicating the presence of mesopores, according to the classification of the International Union of Pure and Applied Chemistry (IUPAC) [13]. Type H_3_ hysteresis loop is usually found on solids with a very wide pore size distribution and type H_4_ corresponds to limited amounts of mesopores limited by micropores. The values obtained of specific surface area (A_BET_) were 24 m^2^/g, 5.20 m^2^/g, and 10.90 m^2^/g for SiO_2_-PEG, SiO_2_-SDS, and SiO_2_-CTAB, respectively (Table 2). Mean pore sizes of 7.07, 8.04, and 8.34 nm were obtained for SiO_2_-PEG, SiO_2_-CTAB, and SiO_2_-SDS samples, respectively, which are similar to the values reported in some works [27,32,33].

### 3.2. Biomimetic Growth of Hydroxyapatite on SiO_2_

The SiO_2_-PEG sample was selected for continuing with the biomimetic growth of hydroxyapatite, biocompatibility tests, and gentamicin loading due to its morphology of fine spheres, with smaller particle size (179 nm), the presence of silanol group (Si–OH) and by its high surface area (24 m^2^/g).

The SiO_2_-PEG sample was immersed for 21 days in SBF. After this time, the biomimetic growth of apatite on SiO_2_-PEG microspheres was studied by FTIR-ATR, X-ray diffraction, SEM-EDS, and TEM techniques. Subsequently, biocompatibility and gentamicin loading capacity of SiO_2_-PEG and SiO_2_-PEG/HAP samples were investigated.

**Figure 4:**
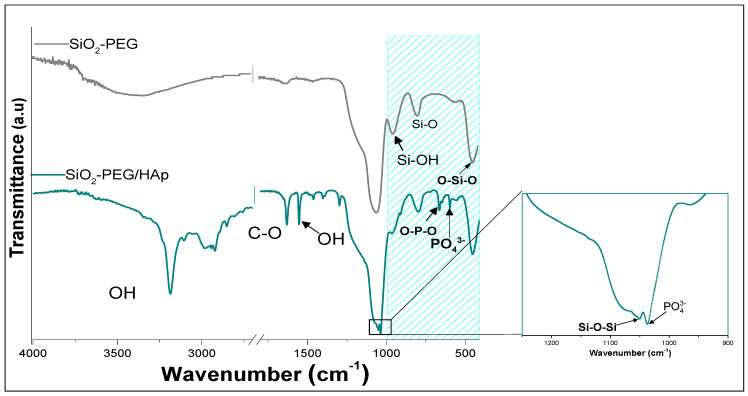
FTIR-ATR spectrum of SiO_2_-PEG before and after the biomimetic growth of hydroxyapatite.

Table 3 indicates the characteristic absorption bands of carbonated hydroxyapatite [32,33,34,35,36,37]. In Figure 4, FTIR spectra of SiO_2_-PEG (before bioactivity induction) are presented for comparative purposes. Spectra of SiO_2_-PEG after inducing biomimetic growth of HAp (SiO_2_-PEG/HAp) evidenced the characteristic bands P–O at 1250 at 400 cm^−1^; moreover, two bands were seen at 3192 cm^−1^ and 2971 cm^−1^, associated with OH, present in the structure of HAp [26,27]. Around 1630 cm^−1^, the C–O bond assigned to the carbonate was observed and at 650 cm^−1^, the bending of the O–P–O bond was observed, characteristic signals of HAp, and the band at 650 cm^−1^ was attributed to the vibration of PO_4_^3−^ [37]. This suggests after immersing SiO_2_ samples in SBF for 21 days, the growth of HAp was obtained on the surface of SiO_2_-PEG.

**Figure 5:**
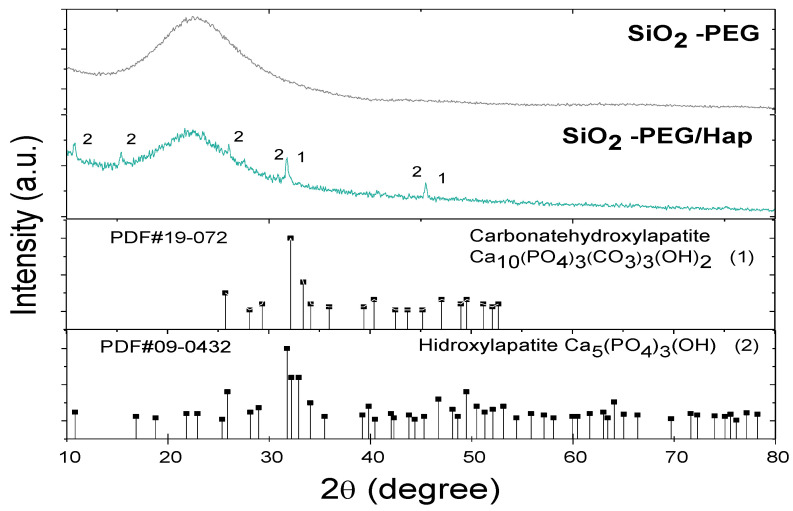
X-ray diffractogram of the SiO_2_-PEG before and after being submerged in the simulated body fluid (SBF) solution for 21 days.

Diffractograms of SiO_2_-PEG and SiO_2_-PEG/HAp samples (Figure 5) showed a characteristic broad peak corresponding to amorphous phase of SiO_2_, which was obtained around 22°. After biomimetic growth (SiO_2_-PEG/HAp sample), small fine peaks were observed, in addition to the broad peak attributed to SiO_2_. The SiO_2_-PEG/HAp sample presented the characteristic diffraction peaks of hydroxyapatite (diffraction pattern PDF # 40-0008). Also, the characteristic diffraction peak of carbonated hydroxyapatite, according to the diffraction pattern PDF # 19-0272, at 31.83° with diffraction plane (1,1,2), is observed. This same peak also corresponds to hydroxyapatite.

**Figure 6:**
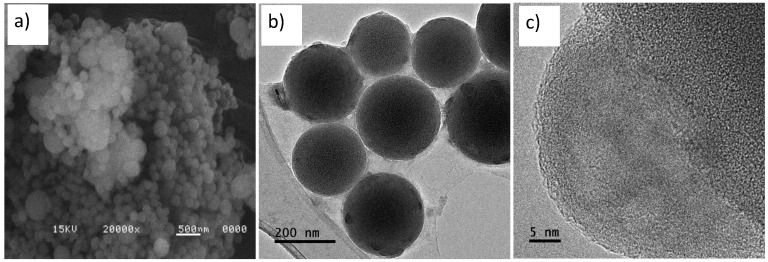
Micrograph of scanning electron microscopy (SEM) at 20,000 X of (**a**) SiO_2_-PEG /HAp, (**b**,**c**) transmission electron microscopy (TEM) of SiO_2_-PEG/HAp.

Figure 6 shows the SEM and TEM micrograph for SiO_2_-PEG/HAp. Various groups of aggregates were observed, wrapped in a continuous layer of HAp (Figure 6a,b), this wrapper is favored by calcium and sodium ions, which allow nucleation of HAp crystals, as cited in the literature [7,10,11,12,38,39,40]. This nucleation and growth of crystals was observed by TEM, in Figure 6c, HAp crystals are observed on the surface of the SiO_2_ sphere. Finally, it should be noted that there was no appreciable change in the spherical morphology of the silica, in the presence of HAp.

The SEM-EDX and TEM studies performed on the SiO_2_-PEG/HAp sample confirm the growth of hydroxyapatite on SiO_2_ spheres.

**Figure 7:**
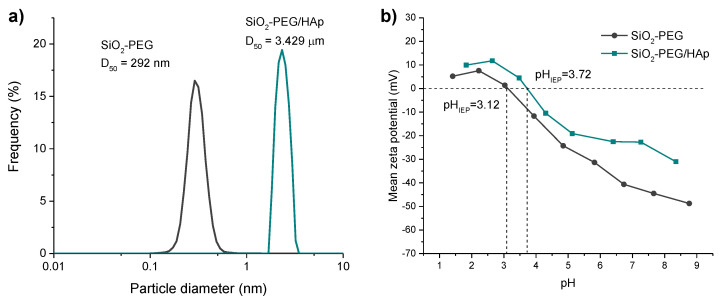
Dynamic light scattering (DLS) spectra (**a**) and zeta potential (**b**) of SiO_2_-PEG and SiO_2_-PEG/HAp samples.

The particle size distribution and the mean zeta potential of SiO_2_-PEG and SiO_2_-PEG/HAp samples were determined by the dynamic light scattering (DLS) and by microelectrophoresis techniques, respectively. Figure 7a shows that the growth of HAp on the SiO_2_ microspheres is accompanied by a significant increase in the average particle size, increasing from 292 nm to 3.429 µm. Figure 7b shows that the pH value of the isoelectric point (pH_IEP_) of the SiO_2_-PEG/HAp sample is 3.72 and it is slightly higher than that of the SiO2-PEG sample (pH_IEP_ = 3.12).

**Figure 8:**
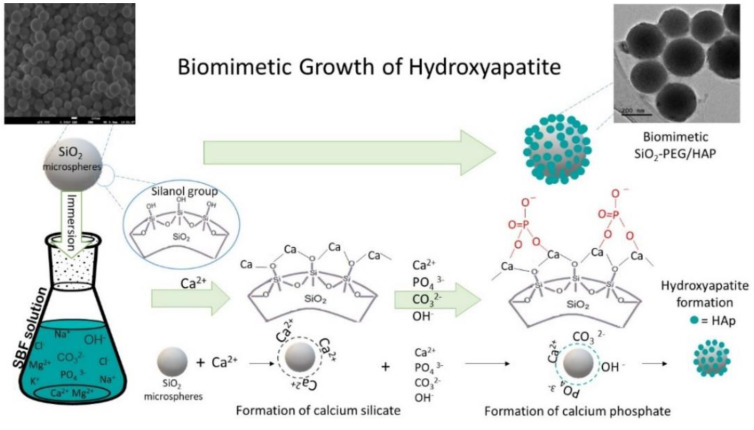
Illustration of the biomimetic growth of HAp on the surface of the silica microspheres.

Based on all results obtained from chemical, structural, and morphological characterization of the SiO_2_-PEG/HAp sample, the biomimetic nucleation and growth of carbonated hydroxyapatite crystals on the surface of SiO_2_ microspheres (SiO_2_-PEG sample) is illustrated in Figure 8.

### 3.3. In Vitro Biocompatibility

#### 3.3.1. Hemolysis Test

**Figure 9:**
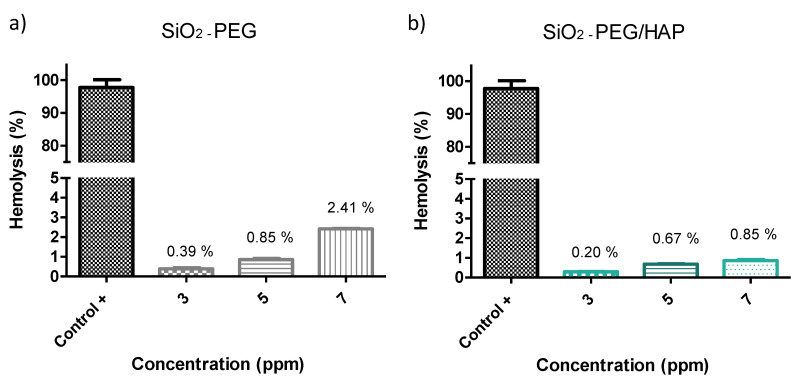
Percentage of hemolysis of (**a**) SiO_2_-PEG and (**b**) SiO_2_-PEG/HAp samples.

A hemolysis test was performed to evaluate the blood biocompatibility of silica spheres, before and after inducing biomimetic growth, this test was carried out following ASTM F756-13 [21], which is specified as hemolytic (%H > 5), slightly hemolytic (5 > %H > 2) and non-hemolytic (2 > %H > 0), is acceptable as biomaterials in percentages lower than 5%. This practice is intended to evaluate the acute in vitro hemolytic properties of materials intended for use in contact with blood; this practice consists of a protocol for a hemolysis test under static conditions with either an extract of the material or direct contact of the material with blood. It was found that SiO_2_-PEG sample evaluated at 3, 5, and 7 ppm show percentages of hemolysis of 0.39%, 0.85%, and 2.41% (Figure 9a). On the other hand, it was found that the SiO_2_-PEG/HAp samples evaluated at 3, 5, and 7 ppm show percentages of hemolysis of 0.20%, 0.67%, and 0.85% (Figure 9b). These results suggest that both samples are non-hemolytic and that the biomimetic growth of HAp on the SiO_2_ spheres enhances biocompatibility by decreasing the percentages of hemolysis. These results were corroborated by the analysis of variance of a single factor (ANOVA), resulting in there being no significant difference between the percentages of hemolysis, obtaining a value of *p* < 0.0001 [13].

#### 3.3.2. Cytotoxicity Using the Cell Line 3T3: MTT Test

**Figure 10:**
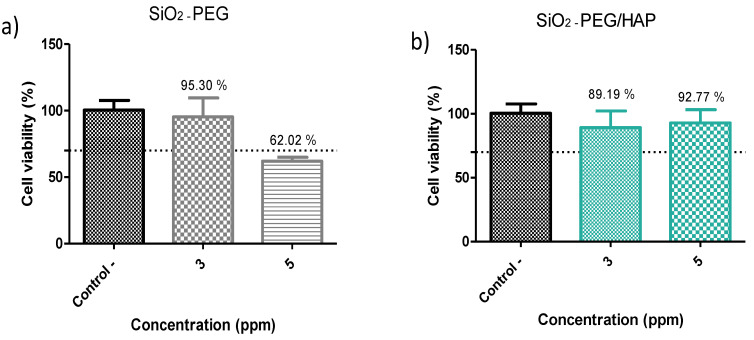
Percentage of cell viability of (**a**) SiO_2_-PEG and (**b**) SiO2-PEG/HAp samples, cultured in 3T3 cells during 24 h of exposure, evaluated at 3 and 5 ppm of sample concentration.

To evaluate biocompatibility, the MTT test was used. It is a colorimetric study based on the capacity of cellular mitochondrial dehydrogenase to reduce the yellow tetrazolium salt to purple formazan crystals. The mouse fibroblast cell line 3T3 was used. All data are presented with mean ± SD, with *n* = 3. The cell viability percentages obtained are shown in Figure 10. Before biomimetic growth, SiO_2_ spheres (SiO_2_-PEG sample) showed percentages considered acceptable of 93.5% for a concentration of 3 ppm, however, when increasing the concentration at 5 ppm, cell viability decreased drastically to 62% (Figure 10a). This effect was not observed in the SiO_2_-PEG/HAp sample, by increasing the concentration from 3 to 5, cellular viability values of 93.5% and 92.34%, respectively, were obtained (Figure 10b). The ANOVA analysis did not show significant differences between the concentrations and the control (*p* < 0.0001 for all samples. Therefore, the biomimetic growth of HAp helps to improve cell viability of SiO_2_ microspheres. Non-cytotoxicity in SiO_2_-PEG/HAp could be related to calcium and phosphate ions, from the growth of HAp in SiO_2_ microspheres. Xu et al. 2005, mention that calcium and phosphate ions are released in the medium, added to the HAp layer, similar to the porosity and structure of a bone that surrounds the SiO_2_ spheres, allowing the formation of bonds with the cells of the osteoblasts of the 3T3 cell line 3T3 [38].

### 3.4. Gentamicin Loading Capacity

**Figure 11:**
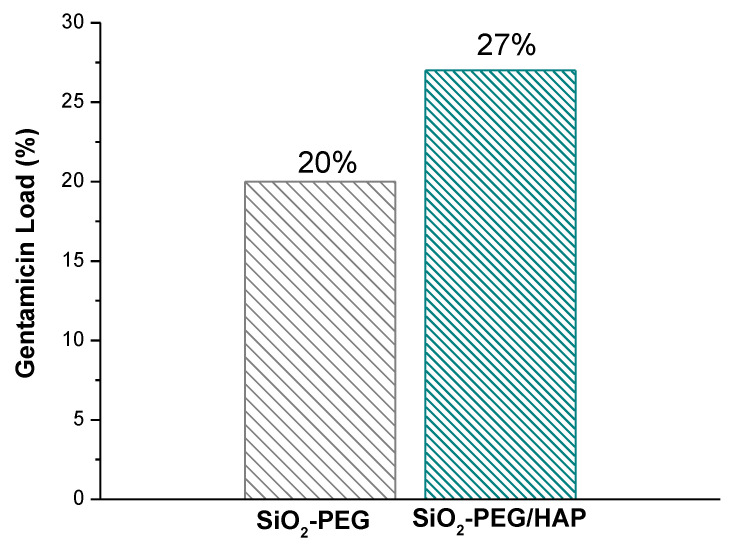
Gentamicin loading capacity of SiO_2_-PEG and SiO_2_-PEG/HAp samples.

Additionally, the gentamicin load was carried out in the SiO_2_ samples before and after the biomimetic growth of HAp to evaluate its loading capacity. Figure 11 shows that gentamicin loading capacity was 20% and 27% for SiO_2_-PEG and SiO_2_-PEG/HAp, respectively. The high loading capacity of gentamicin in the SiO_2_-PEG/HAp sample is attributed to the fact that the hydroxyapatite layer that surrounds the SiO_2_ spheres contributes to the sorption of gentamicin molecules.

Before using SiO_2_ nanoparticles in biomedical applications, they must have several requirements; for example, the particle size must be less than 150 nm if they are to be used intravenously. It is also desirable that SiO_2_ dispersions in physiological fluids are stable in order to optimize their efficiency [41]. Varache et al. (2015) prepared a highly stable coloidal suspension of SiO_2_ nanoparticles of MCM-41, using NaOH solution free of carbonate and using a N_2_ constant flow [42]. In addition to these two requirements, it is desirable that the porous structure of SiO_2_ nanoparticles remains intact for a long time. Burleigh et al. (2003) reported that mesoporous SiO_2_ (SBA-15) stored for 10 months did not have structural changes because they have thick pore walls that are, thus, stable to water induced hydrolysis [43]. On the other hand, Broyer et al. (2002) reported that pore volume of calcined MCM-41 was decreased after aging during 3 months [44]. Adeniran and Mokaya carried investigated the structural stability of freshly and 12-year-old MCM-41 samples; they reported that calcined MCM-41 samples retain their structural properties over a period of 12 years [45].

## 4. Conclusions

The use of PEG surfactant as a template in SiO_2_ synthesis by the modified Stöber method allowed a sample formed by SiO_2_ microspheres to be obtained, with an average particle size of 179 nm. The heat treatment was considered undesirable because the FTIR-ATR spectra showed the elimination of the silanol group (Si–OH) in the SiO_2_ samples. Also, the heat treatment caused the collapse of the spherical morphology of SiO_2_ microparticles. The biomimetic growth of hydroxiapatite in the SiO_2_ spheres was carried out successfully; characteristic absorption bands of HAp were observed (P–O, O–P–O, and vibrations of the PO_4_^3−^ group) in the FTIR-ATR spectrum of the SiO_2_-PEG/HAp sample. Diffraction peaks of this sample are attributed to HAp and carbonated hydroxyapatite. Similarly, SEM-EDX and TEM studies confirm the formation of HAp crystals on the surface of the SiO_2_ microspheres. Regarding in vitro biocompatibility, the hemolysis test suggests that after the growth of HAp, the biocompatibility of SiO_2_ microspheres is favored. In the same way, the results of cell viability suggest that the biomimetic growth of HAp over SiO_2_ microspheres results in percentages greater than 90% of viability of 3T3 cells for the different concentrations analyzed, it being considered as a non-cytotoxic material. This work demonstrates a clear improvement in the biocompatibility and gentamicin-loading capacity of SiO_2_ spheres containing HAp biomimetically grown, which indeed opens the door to further studies in the use of SIO_2_-PEG/HAp as a system for drug loading and delivery. Further experiments regarding load and delivery of other drugs are required.

## Figures and Tables

**Table 1:** Characteristic absorption bands of SiO_2_.

Band (cm^−1^)	Assignation
3000–3500	ν O–H
2937, 2985, 1446 and 1392	CH_3_ and CH_2_ groups
1049	ν Si–O–Si
962 and 567	ν Si–OH
803	ν O–Si–O
465	δ Si–O

Vibratory mode: δ = in flat bending; ν = stretching.

**Table 2:** Brunauer–Emmet–Teller (BET) and Barret–Joyner–Halenda (BJH) analysis results of SiO_2_-CTAB, SiO_2_-SDS, and SiO_2_-PEG samples.

Parameter	SiO_2_-PEG	SiO_2_-CTAB	SiO_2_-SDS
Specific surface area (m^2^/g)	24.00	5.20	10.90
Mean pore size (nm)	7.07	8.09	8.34
Pore volume (cm^3^/g)	0.04	0.02	0.01

**Table 3:** Characteristics absorption bands of carbonated hydroxyapatite [32,33,34,35,36,37,38].

Band (cm^−1^)	Assignation
3000–3500	ν O-H
1415–1500 y 875	ν C-O de CO_3_^2−^
1080–1095 y 953	ν P-O
670	ν PO_4_^3−^
606, 567 y 468	δ O-P-O

Vibratory mode: δ = in flat bending; ν = stretching.

## Data Availability

Data presented in this study are available on request from the corresponding author.

## References

[1] Farid S.B.H. (2019). Bioceramics: For Materials Science and Engineering.

[2] Riggs B.L., Khosla S., Melton L.J. (2002). Sex steroids and the construction and conservation of the adult skeleton. Endocr. Rev..

[3] Bharadwaz A., Jayasuriya A.C. (2020). Recent Trends in the application of widely used natural and synthetic polymer nanocomposites in bone tissue regeneration. Mater. Sci. Eng. C.

[4] Rufino-Senra M., Barbosa de Lima R., Saboya-Souza D.H., Vieira-Marques M.F., Neves-Monteiro S. (2020). Thermal characterization of hydroxyapatite or carbonated hydroxyapatite hybrid composites with distinguished collagens for a bone graft. J. Mater. Res. Technol..

[5] Salimi S. (2020). Functionally graded calcium phosphate bioceramics: An overview of preparation and properties. Ceram. Int..

[6] Bystrova A.V., Dekhtyar Y.D., Popov A.I., Coutinho J., Bystrov V.S. (2015). Modified Hydroxyapatite structure and properties: Modeling and synchrotron data analysis of modified hydroxyapatite structure. Ferroelectrics.

[7] Beck G.R., Ha S.W., Camalier C.E., Yamaguchi M., Li Y., Lee J.K., Weitzmann M.N. (2012). Bioactive silica-based nanoparticles stimulate bone-forming osteoblasts, suppress bone-resorbing osteoclasts, and enhance bone mineral density in vivo. Nanomed. Nanotechnol. Biol. Med..

[8] Santin M., Phillips G., Santin M., Phillips G. (2012). History of biomimetic, bioactive, and bioresponsive biomaterials. Biomimetic, Bioresponsive, and Bioactive Materials: An Introduction to Integrating Materials with Tissues.

[9] Yilmaz B., Pazarceviren A.E., Tezcaner H., Evis Z. (2020). Historical development of simulated body fluids used in biomedical applications: A review. Microchem. J..

[10] Vallés-Lluch A., Gallego-Ferrer G., Monleón-Pradas M. (2009). Biomimetic apatite coating on P(EMA-co-HEA)/SiO_2_ hybrid nanocomposites. Polymers.

[11] Shi S., Goto T., Cho S.H., Sekino T. (2000). Surface-morphology modification of ceramic-based composites for a photocatalytic activity via simple chemical and heat treatments. J. Ceram. Soc. Jpn.

[12] Catauro M., Bollino F., Papale F., Gallicchio M., Pacifico S. (2015). Influence of the polymer amount on bioactivity and biocompatibility of SiO_2_/PEG hybrid materials synthesized by sol-gel technique. Mater. Sci. Eng. C.

[13] Moussa M., Mankoci S., Ustriyana P., Zhang N., Abdelmagid S., Molenda J., Murphy W.L., Safadi F.F., Sahai N. (2016). Orthosilicic acid, Si(OH)_4_, stimulates osteoblast differentiation in vitro by upregulating miR-146a to antagonize NF-κB activation. Acta Biomater..

[14] Mehra R.R., Tiwari P., Basu A., Duttkonar A. (2019). Correction: In search of bioinspired hydrogels from amphiphilic peptides: A template for nanoparticle stabilization for the sustained release of anticancer drugs. New J. Chem..

[15] Wu C., Chang J., Fan W. (2012). Bioactive mesoporous calcium-silicate nanoparticles with excellent mineralization ability, osteostimulation, drug-delivery and antibacterial properties for filling. J. Mater. Chem..

[16] Kurdyukov D.A., Eurov D.A., Kirilenko D.A., Sokolov V.V., Golubev V.G. (2018). Tailoring the size and microporosity of Stöber silica particles. Microporous Mesoporous Mater..

[17] Lei Y., Chen X., Song H., Hu Z., Cao B. (2017). The influence of thermal treatment on the microstructure and thermal insulation performance of silica aerogels. J. Non. Cryst. Solids.

[18] Ríos F., Fernández-Arteaga A., Fernández-Serrano M., Jurado E., Lechuga M. (2018). Silica micro- and nanoparticles reduce the toxicity of surfactant solutions. J. Hazard. Mater..

[19] Ibarra-Alonso M.C., Reyna-Martínez R., Narro Céspedes R.I., Reyes Acosta Y.K., Martínez-Luevanos A., Zugasti-Cruz A., Neira-Velázquez M.G., Sánchez-Valdés S., Soria-Arguello G. (2020). Effect of thermal and argon plasma treatment in SiO_2_ spheres, assessing the effectiveness in the elimination of organic waste. Rev. Mex. Ing. Química.

[20] Kokubo T., Takadama H. (2006). How useful is SBF in predicting in vivo bone bioactivity. Biomaterials.

[21] ASTM F756-13 (2014). Standard Practice for Assessment of Hemolytic Properties of Materials. https://www.astm.org/Standards/F756.htm.

[22] 10993–5:2009 (2009). International Organization for Standardization. Biological Evaluation of Medical Devices—Part 5: Tests for In Vitro Cytotoxicity. https://www.iso.org/standard/36406.html.

[23] Travaglini L., Picchetti P., Del Giudice A., Galantini L., De Cola L. (2019). Tuning and controlling the shape of mesoporous silica particles with CTAB/sodium deoxycholate catanionic mixtures. Microporous Mesoporous Mater..

[24] Wiercigroch-Walkosz K., Cichos J., Karbowiak M. (2019). Growth of silica shell on hydrophobic upconverting nanocrystals—Mechanism and control of porosity. Colloids Surf. A Physicochem. Eng. Asp..

[25] Raju M., van Duin A.C.T., Fichthorn K. (2014). Mechanisms of Oriented Attachment of TiO_2_ Nanocrystals in Vacuum and Humid Environments: Reactive Molecular Dynamics. Nano Lett..

[26] Gholizadeh R., Wang Y. (2018). Molecular dynamics simulation of the aggregation phenomenon in the late stages of silica materials preparation. Chem. Eng. Sci..

[27] Ching-Hung L., Yi-Ming S. (2017). Influence of the surface properties of nano-silica on the dispersion and isothermal crystallization kinetics of PHB/silica nanocomposites. Mater. Chem. Phys..

[28] Liu Y., Tourbin M., Lachaize S., Guiraud P. (2013). Silica nanoparticles separation from water: Aggregation by cetyltrimethylammonium bromide (CTAB). Chemosphere.

[29] Guo-Yong H., Sheng-Ming X., Lin-Yan L., Xue-Jun W. (2014). Effect of surfactants on dispersion property and morphology of nano-sized nickel powders. Trans. Nonferrous Met. Soc. China.

[30] He S., Huang Y., Chen G., Feng M., Dai H., Yuan B., Chen X. (2010). Effect of heat treatment on hydrophobic silica aerogel. J. Am. Chem. Soc..

[31] Lin Y., Haynes C.L. (2010). Impacts of Mesoporous Silica Nanoparticle Size, Pore Ordering, and Pore Integrity on Hemolytic Activity. J. Am. Chem. Soc..

[32] Yu H., Zhang H., Wang X., Gu Z., Li X., Deng F. (2007). Local structure of hydroxy—Peroxy apatite: A combined XRD, FT-IR, Raman, SEM, and solid-state NMR study. J. Phys. Chem. Solids.

[33] Londoño M.E., Echavarría A., La Calle F.D. (2006). Características cristaloquímicas de la hidrixiapatita sintética tratada a diferentes temperaturas. Esc. Ing. Antioq..

[34] Siriphannon P., Kameshima Y., Yasumori A., Okada K., Hayashi S. (2002). Formation of hydroxyapatite on CaSiO_3_ powders in simulated body fluid. J. Eur. Ceram. Soc..

[35] Angelopoulou A., Efthimiadou E.K., Kordas G., Angelopoulou E.K. (2015). Efthimiadou. A new approach to fabricate bioactive silica binary and ternary hybrid microspheres. Mater. Sci. Eng. C.

[36] Li B., Luo W., Wang Y., Wu H., Zhang C. (2016). Bioactive SiO_2_-CaO-P_2_O_5_ hollow nanospheres for drug delivery. J. Non. Cryst. Solids.

[37] Fisichella M., Dabboue H., Bhattacharyya S., Saboungi M.L., Salvetat J.P., Hevor T., Guerin M. (2009). Mesoporous silica nanoparticles enhance MTT formazan exocytosis in HeLa cells and astrocytes. Toxicol. Vitr..

[38] Xu H.H.K., Simon C.G. (2005). Fast setting calcium phosphate-chitosan scaffold: Mechanical properties and biocompatibility. Biomaterials.

[39] Tamanna T., Bulitta J.B., Yu A. (2015). Controlling antibiotic release from mesoporous silica nano-drug carriers via self-assembled polyelectrolyte coating. J. Mater. Sci. Mater. Med..

[40] Ji J., Hao S., Wu D., Huang R., Xu Y. (2011). Preparation, characterization and in vitro release of chitosan nanoparticles loaded with gentamicin and salicylic acid. Carbohydr. Polym..

[41] Cebrián V., Yagüe C., Arruebo M., Martín-Saavedra F.M., Santamaría J., Vilaboa N. (2011). On the role of the colloidal stability of mesoporous silica nanoparticles as gene delivery vectors. J. Nanopart. Res..

[42] Varache M., Bezverkhyy I., Bouyer F., Chassagnon R., Baras F., Bouyer F. (2015). Improving structural stability of water-dispersed MCM-41 silica nanoparticles through post-synthesis pH aging process. J. Nanopart. Res..

[43] Burleigh M.C., Markowitz M.A., Jayasundera S., Spector M.S., Thomas C.W., Gaber B. (2003). Mechanical and hydrothermal stabilities of aged periodic mesoporous organosilicas. J. Phys. Chem. B.

[44] Broyer M., Valange S., Bellat J.P., Bertrand O., Weber G., Gabelica Z. (2002). Influence of aging, thermal, hydrothermal, and mechanical treatments on the porosity of MCM-41 mesoporous silica. Langmuir.

[45] Adeniran B., Mokaya R. (2012). On the Shelf Life and Aging Stability of Mesoporous Silica: Insights on Thermodynamically Stable MCM-41 Structure from Assessment of 12-Year-Old Samples. Chem. Mater..

